# Incidence, Stage, Treatment, and Survival of Noncardia Gastric Cancer

**DOI:** 10.1001/jamanetworkopen.2023.30018

**Published:** 2023-08-21

**Authors:** Merel J. M. van Velzen, Michelle Braemer, Grard A. P. Nieuwenhuijzen, Johanna W. van Sandick, Peter D. Siersema, Jelle P. Ruurda, Marcel Verheij, Manon C. W. Spaander, Laurens V. Beerepoot, Nadia Haj Mohammad, Hanneke W. M. van Laarhoven, Rob H. A. Verhoeven

**Affiliations:** 1 Medical Oncology, Amsterdam UMC, University of Amsterdam, Amsterdam, the Netherlands; 2 Cancer Center Amsterdam, Cancer Treatment and Quality of Life, Amsterdam, the Netherlands; 3 Department of Surgery, Catharina Hospital, Eindhoven, Netherlands; 4 Department of Surgery, Antoni van Leeuwenhoek–Netherlands Cancer Institute, Amsterdam, Netherlands; 5 Department of Gastroenterology and Hepatology, Radboud Institute for Health Sciences, Radboud University Medical Centre, Nijmegen, the Netherlands; 6 Department of Surgery, University Medical Center Utrecht, Utrecht University, Utrecht, the Netherlands; 7 Department of Radiation Oncology, Radboud University Medical Center, Nijmegen, the Netherlands; 8 Department of Radiation Oncology, Netherlands Cancer Institute, Amsterdam, the Netherlands; 9 Department of Gastroenterology and Hepatology, Erasmus MC University Medical Center, Rotterdam, the Netherlands; 10 Department of Medical Oncology, Elisabeth Tweesteden Ziekenhuis, Tilburg, the Netherlands; 11 Department of Medical Oncology, University Medical Center Utrecht, Utrecht University, Utrecht, the Netherlands; 12 Department of Research and Development, Netherlands Comprehensive Cancer Organisation, Utrecht, the Netherlands

## Abstract

**Importance:**

Gastric cancer is the fifth most common cancer worldwide, and investigating its incidence, characteristics, treatment, and outcomes over the past decades can help in selecting clinical strategies and future research directions.

**Objective:**

To analyze the trends in incidence, staging, and treatment of gastric cancer.

**Design, Setting, and Participants:**

This nationwide, population-based cohort study included patients diagnosed with noncardia gastric cancer (NCGC) between 1989 and 2021 in the Netherlands.

**Main Outcomes and Measures:**

Differences in tumor characteristics, treatment, and survival were analyzed per fixed time periods (1989-1993, 1994-1998, 1999-2003, 2004-2008, 2009-2013, 2014-2018, and 2019-2021).

**Results:**

In total, 47 014 patients (median [IQR] age, 73 [64-80] years; 28 032 [60%] male patients) were identified with mostly adenocarcinomas of the antrum region (when location was known). Age-standardized incidence decreased from 20.3 to 6.1 per 100 000 person-years between 1989 and 2021. During the study period, unknown T and N stages were recorded less frequently, and metastatic disease was diagnosed more frequently (1989-1993: 2633 of 9493 patients [28%]; 2019-2021: 1503 of 3200 patients [47%] in 2019-2021). Over time, fewer patients with metastatic disease underwent surgery with or without other treatment modalities (68% in 1989-1993 vs 64% in 2019-2021), and palliative chemotherapy in metastatic NCGC increased from 9% to 40%. For patients with nonmetastatic disease, 5-year relative survival improved from 28% (95% CI, 26.5%-29.2%) to 36% (95% CI, 33.5%-37.6%) between 1989 and 2021. For patients with nonmetastatic disease undergoing a resection, 5-year survival increased from 40% (95% CI, 38.3%-41.8%) to 51% (95% CI, 47.9%-53.3%). For patients with metastatic disease, 1-year relative survival increased from 10% (95% CI, 8.7%-11.1%) to 19% (95% CI, 17.2%-21.6%), but 3-year relative survival remained poor at 5% (95% CI, 3.6%-7.5%).

**Conclusions and Relevance:**

In this nationwide cohort study involving 47 014 patients diagnosed with NCGC (1989-2021), the results showed a decrease in incidence, more accurate staging, a shift in treatment modalities, and improved patient survival.

## Key Points

**Question:**

Are there differences in incidence, tumor characteristics, treatment, and survival of noncardia gastric cancer (NCGC) in the period 1989 to 2021?

**Findings:**

This cohort study including 47 014 patients with NCGC between 1989 and 2021 found a significant decrease in NCGC incidence, more accurate staging, a shift in treatment modalities, and improved overall survival.

**Meaning:**

Despite a decrease in incidence and improved outcomes, NCGC is still among the most lethal cancers, and thus, future research is warranted to discover more advantageous treatments.

## Teaser

This cohort study analyzes the trends in incidence, tumor characteristics, staging, and treatment of noncardia gastric cancer among patients in the Netherlands from 1989 to 2021.

## Introduction

Gastric cancer is currently the fifth most frequently diagnosed cancer and the fourth leading cancer-related cause of death worldwide.^1^ Especially in Eastern and Central Asia and Latin and South America, incidence rates are high, while the lowest incidence rates are seen in Northern America and Africa. While an increase in the incidence of esophageal cancer has been observed in Western Europe over the past decades, the opposite is true for gastric cancer, being diagnosed less often over the years.^1^ This decline in incidence rate has been attributed to dietary changes and the use of the refrigerator, together with increasing awareness of the risks of *Helicobacter pylori* infections, leading to more frequent treatment and thus reduced prevalence of the bacterium.^2^

Cancers arising in the cardia, the region closest to the gastroesophageal junction, often share similar epidemiological and etiological features with distal esophageal adenocarcinomas and therefore are usually regarded as a separate entity compared with noncardia gastric cancers (NCGC).^3^ Different attempts have been made to classify NCGC based on histology, of which the Laurén classification and World Health Organization classification are most widely used.^4,5,6^ Next to anatomical and histological classification, molecular characterization, such as that determined by The Cancer Genome Atlas, divides gastric cancer in 4 subtypes.^7,8^

Despite its poor prognosis, treatment of both localized and advanced NCGC has changed over the past 30 years, mostly in the field of multimodal treatments. Prior to the first Dutch gastric cancer guideline published in 2009, no national evidence-based guidelines were available for the treatment of gastric cancer. Three landmark studies have changed clinical practice. In 2001, the SWOG-INT0116 study demonstrated a survival benefit by adding postoperative chemoradiotherapy.^9^ In 2006, the Medical Research Council Adjuvant Gastric Infusional Chemotherapy (MAGIC) trial was the first to show an important survival benefit of perioperative chemotherapy for patients with localized gastric cancer.^10^ Subsequently, in 2018 the FLOT regimen (5FU, leucovorin, oxaliplatin, and docetaxel) was proven superior to perioperative chemotherapy using epirubicine, cisplatinum, and capecitabine and is now the preferred treatment in Western countries.^10,11^ Additionally, surgical techniques have improved, and centralization of highly specialized (minimally invasive) surgical procedures has led to improved cancer-specific survival and less mortality.^12^ Development in the diagnostic workup of patients, eg, by implementing diagnostic laparoscopy to detect otherwise occult peritoneal metastasis, has led to improved tumor staging and treatment accordingly.^13^

When investigating tumor-specific trends and population-based outcomes of different treatments in various cancer types, cancer registries have shown to be invaluable.^14,15^ In NCGC, previous studies have described the changes in epidemiologic factors, but information on treatment and survival estimates are often missing.^16,17,18^ For this study, we used information from the Netherlands Cancer Registry (NCR) to study trends in incidence, stage, treatment strategies, and survival among patients with NCGC in the Netherlands from 1989 to 2021.

## Methods

### Data Collection and Patient Selection

For this retrospective cohort study, we selected patients who were diagnosed with NCGC between 1989 and 2021 from the NCR.^19^ Topography and morphology of the primary tumor were coded according to the *International Classification of Diseases for Oncology, third edition* (ICD-O-3).^20^ All patients diagnosed with NCGC (*ICD-O-3* topography codes, C16.1-C16.9) were included (not included were patients with gastrointestinal stroma cell tumors, sarcomas, and lymphomas), and patients with neuro-endocrine tumors (NEC) were excluded. Median follow-up was calculated using the reversed Kaplan-Meier method and reported in years.

The NCR is a nationwide, population-based cancer registry that includes all patients diagnosed with cancer in the Netherlands and is directly linked to the Dutch Nationwide Pathology Database (PALGA) that comprises all histologically confirmed cancer diagnoses. Data managers of the Netherlands Comprehensive Cancer Organisation (IKNL) are trained to extract information regarding cancer diagnosis, tumor stage, and patient and treatment characteristics from the patient’s medical records. According to the Central Committee on Research involving Human Subjects, this type of study did not require approval from an ethics committee in The Netherlands. This study was approved by the Privacy Review Board of the NCR and the scientific committee of the Dutch Upper GI Cancer Group and follows the Strengthening the Reporting of Observational Studies in Epidemiology (STROBE) reporting guideline.

Since the NCR was initiated in 1989, various *TNM Classifications of Malignant Tumours* (TNM), published by the Union of International Cancer Control, have been used. To ensure similar stage groups over time in the current study, all stages were recoded according to the fifth edition.^21^ Due to strict coding regulations in the NCR prior to 2010, patients treated as having clinical M0 (cM0) were sometimes registered as cMx. As per 2010, coding regulations on M status became more flexible, which resulted in almost no patients being registered as cMx, with an accompanying increase in cM0 stages but not in cM1 stages. Therefore, we decided to register all patients registered as cMx as cM0.^22^

### Classification of Treatment

First, we categorized treatment for nonmetastatic NCGC into 6 treatment groups, ie, no tumor treatment, systemic or local treatment but no surgery, surgery alone (including endoscopic resections), chemotherapy alone, chemotherapy and surgery, and surgery combined with other (local or systemic) treatments. For patients with nonmetastatic disease undergoing surgery, we also considered the timing of chemotherapy, resulting in 4 additional treatment groups, ie, surgery with no chemotherapy, surgery with neoadjuvant chemotherapy, surgery with adjuvant chemotherapy, or surgery with perioperative chemotherapy. For patients with metastatic NCGC, we investigated 4 treatment groups, ie, no surgery and no chemotherapy, surgery without chemotherapy, chemotherapy without surgery, and chemotherapy and surgery. Because of the relatively limited role of radiotherapy and chemoradiotherapy in the treatment of NCGC, it was not described separately but was covered by other treatments.

### Statistical Analysis

The study period between 1989 and 2021 was divided into 7 time periods: 1989 to 1993, 1994 to 1998, 1999 to 2003, 2004 to 2008, 2009 to 2013, 2014 to 2018, and 2019 to 2021. Patient characteristics were displayed in counts and percentages per period, and a χ^2^ test was used to evaluate the statistical significance of differences between the periods.

Incidence rates were age standardized to European standard population and defined as the number of new patients per 100 000 inhabitants per year. To assess changes in incidence rates, we used joinpoint software to calculate the estimated annual percentage change (EAPC), which depicts the annual change in age-standardized incidence rate over multiple years.^23^ Relative survival was estimated using the Pohar Perme method for estimating net survival, using an expected mortality rate based on the general population according to sex, age, and year of death.^24^ We constructed a multivariable regression model including age, sex, morphology, and Laurén classification to calculate the relative excess risk (RER) of death throughout the study period, corrected for these factors.^25^ *P* values <.05 were considered statistically significant, and all tests were 2-tailed.

## Results

### Patient Characteristics

From the NCR, 47 161 patients (28 032 [60%] male patients) diagnosed with NCGC between 1989 and 2021 were identified, of whom 147 patients with a NEC were excluded. Table 1 shows the patient and tumor characteristics of the remaining 47 014 patients separately for each period. Median (IQR) follow-up was 17.7 (95% CI, 17.2-18.3 years).

**Table 1.** Baseline Characteristics per Period

Characteristic	Patients, No. (%)							*P* value^a^
	1989-1993 (n = 9493)	1994-1998 (n = 8100)	1999-2003 (n = 7337)	2004-2008 (n = 6697)	2009-2013 (n = 6556)	2014-2018 (n = 5631)	2019-2021 (n = 3200)	
Sex								
Male	5688 (60)	4809 (59)	4332 (59)	3966 (59)	3940 (60)	3403 (60)	1894 (59)	.62
Female	3805 (40)	3291 (41)	3005 (41)	2731 (41)	2616 (40)	2228 (40)	1306 (41)	
Age, y								
Median (IQR)	72 (64-80)	73 (64-80)	72 (63-80)	73 (63-80)	73 (64-80)	73 (64-80)	73 (63-80)	.15
≤49	602 (6)	495 (6)	499 (7)	468 (7)	463 (7)	353 (6)	209 (7)	.11
50-64	1895 (20)	1641 (20)	1493 (20)	1367 (20)	1308 (20)	1070 (19)	662 (21)	
65-79	4546 (48)	3795 (47)	3495 (48)	3128 (47)	3024 (46)	2699 (48)	1469 (46)	
≥80	2450 (26)	2169 (27)	1850 (25)	1734 (26)	1761 (27)	1509 (27)	860 (27)	
Tumor location								
Fundus	294 (3)	225 (3)	220 (3)	155 (2)	276 (4)	251 (5)	138 (4)	<.001
Corpus	1140 (12)	1086 (13)	956 (13)	963 (14)	1366 (21)	1346 (24)	901 (28)	
Antrum including pylorus	3131 (33)	2851 (35)	2589 (35)	2407 (36)	2246 (34)	2124 (38)	1248 (39)	
Unknown or overlapping	4928 (52)	3938 (49)	3572 (49)	3172 (47)	2668 (41)	1910 (34)	913 (29)	
Morphology								<.001
Adenocarcinoma	9052 (95)	7783 (96)	7026 (96)	6457 (96)	6294 (96)	5389 (96)	2982 (93)	<.001
Diffuse	1860 (20)	1791 (22)	2015 (28)	2091 (31)	2380 (36)	2229 (40)	1308 (41)	
Intestinal	6056 (64)	5275 (65)	4538 (62)	4022 (60)	3648 (56)	2868 (51)	1444 (45)	
Miscellaneous	1136 (12)	717 (9)	473 (6)	344 (5)	266 (4)	292 (5)	230 (7)	
Other	260 (3)	317 (4)	311 (4)	240 (4)	262 (4)	242 (4)	218 (7)	
Unknown	181 (2)	0	0	0	0	0	0	
cT stage								
cT_0_-cT_is_	34 (0)	17 (0)	14 (0)	20 (0)	29 (0)	36 (1)	30 (1)	<.001
cT_1_	273 (3)	175 (2)	200 (3)	181 (3)	247 (4)	154 (3)	81 (3)	
cT_2_	419 (4)	260 (3)	393 (5)	653 (10)	1897 (29)	2897 (51)	1871 (59)	
cT_3_	505 (5)	407 (5)	347 (5)	388 (6)	232 (4)	261 (5)	228 (7)	
cT_4_	1524 (16)	1232 (15)	1141 (16)	942 (14)	690 (11)	443 (8)	241 (8)	
cT_x_	6738 (71)	6009 (74)	5242 (71)	4513 (67)	3461 (38)	1840 (33)	749 (23)	
cN stage								
cN_0_	1806 (19)	1490 (18)	1559 (21)	1686 (25)	2597 (40)	2519 (45)	1455 (46)	<.001
cN+	1829 (19)	1598 (20)	1798 (25)	2082 (31)	2295 (35)	2275 (40)	1402 (44)	
cN_x_	5858 (62)	5012 (62)	3980 (54)	2929 (48)	1664 (25)	837 (15)	343 (11)	
cM stage								
cM_0_	6860 (72)	5757 (71)	5057 (69)	4445 (66)	4017 (61)	3211 (57)	1697 (53)	<.001
cM_1_	2633 (28)	2343 (29)	2280 (31)	2252 (34)	2539 (39)	2420 (43)	1503 (47)	

^a^ *P* values were calculated using a χ^2^ test.

No significant changes were seen in the distribution of age and sex over time. In 1989 to 1993 most patients had an unknown or overlapping tumor location (4928 of 9493 [52%]), but in 2019 to 2021 most patients had a distal tumor located in the antrum or pylorus (1248 of 3200 [39%]) and 901 patients (28%) had a tumor located in the corpus of the stomach. Over time, the proportion of tumors clinically staged as Tx or T4 decreased, and the proportion of tumors staged as T2 increased. In 1989 to 1993, 5858 patients (62%) had an unknown N stage (N_X_), which was only true for 343 patients (11%) in 2019 to 2021 (*P* < .001) In 1989 to 1993, 28% of patients (2633 patients) were diagnosed with metastatic disease (M_1_), and in 2019-2021, this proportion was 47% (1503 patients) (*P* < .001).

**Figure 1.**
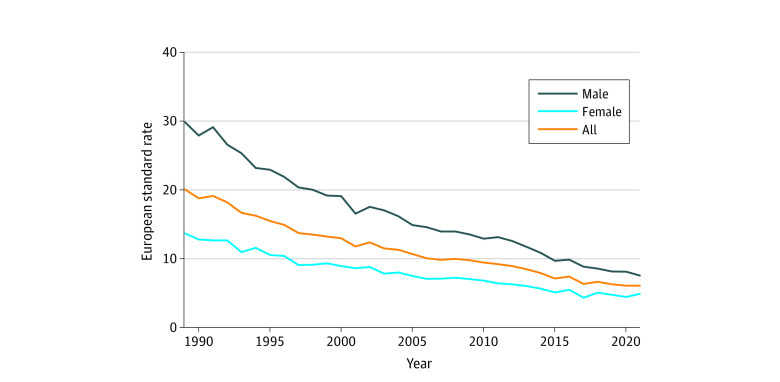
Incidence of Noncardia Gastric Cancer in European Age-Standardized Rate European age-standardized rate is defined as the number of new patients per 100 000 person-years.

Between 1989 and 2021, the European age-standardized incidence of NCGC decreased from 20.3 to 6.1 patients per 100 000 person-years for men and women together. One joinpoint was detected, and the EAPC was −4.3% (95% CI, −5.82% to –2.80%) between 1989 and 1996 and −3.46% (95% CI, −3.68% to –3.23%) between 1996 and 2021. The ESR of men decreased from 29.9 patients per 100 000 person-years in 1989 to 7.52 patients per 100 000 person-years in 2021. One joinpoint was detected, and the EAPC was −3.9% (95% CI, −4.10% to 3.67%) between 1989 and 2013 and −4.9% (95% CI, −5.95% to –3.73%) between 2013 and 2021. The ESR of women decreased from 13.7 to 4.93 patients per 100 000 person-years for women, and no joinpoints were detected, with an EAPC of −3.12% (95% CI, −3.35% to –2.88%) (Figure 1).

### Treatment

**Figure 2.**
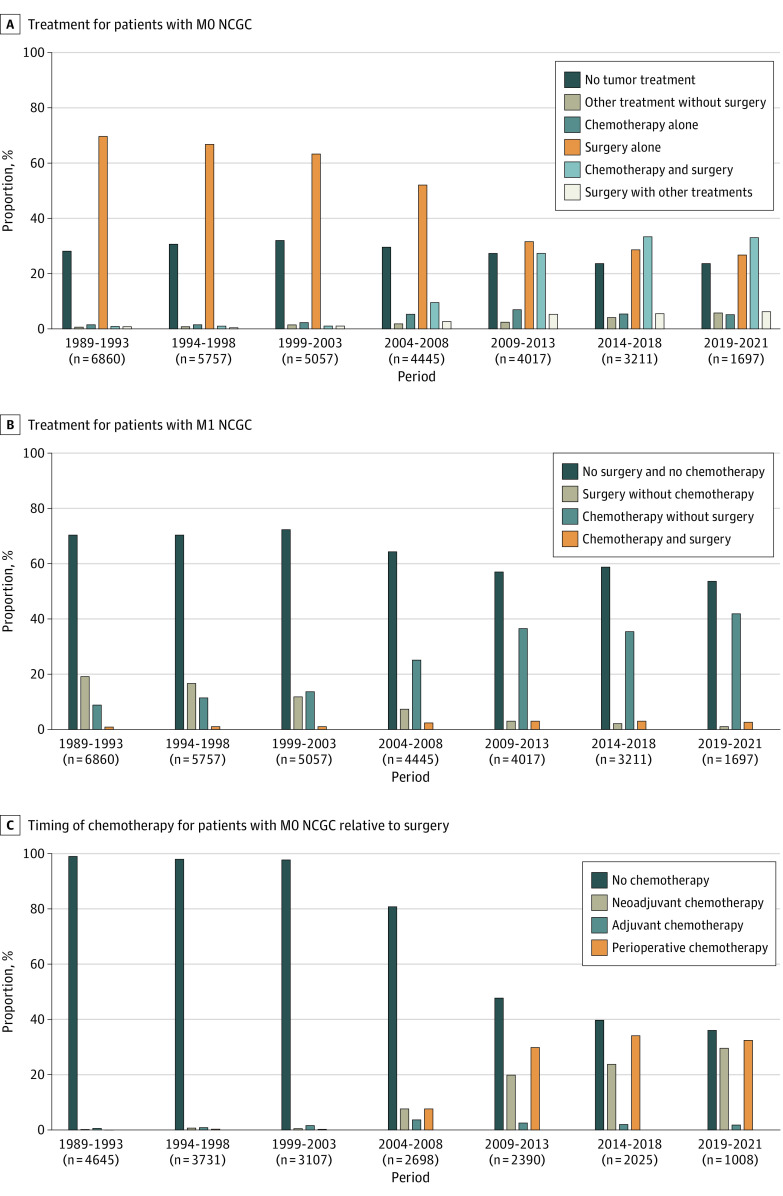
Treatment per Period in Percentages

Between 1989 and 2021, the proportion of patients with nonmetastatic disease who underwent no surgery and no chemotherapy did not significantly change (Figure 2A). The number of patients undergoing surgery decreased slightly (from 4645 of 6860 [68%] in 1989-1993 to 1088 of 1697 [64%] in 2019-2021), and an increase in the use of perioperative and neoadjuvant treatment was observed, starting between 2004 and 2008. However, 364 of 1008 patients (36%) undergoing surgery did not receive multimodality treatment in 2019 to 2021 (Figure 2C).

In 1989 to 1993, only 756 of 2633 patients (29%) with metastatic disease received any type of tumor directed therapy (chemotherapy or surgery), which increased to 649 of 1503 (43%) in 2019 to 2021 (Figure 2B). In 1989 to 1993, 518 patients (20%) with metastatic disease were treated with surgery (with or without chemotherapy), whereas only 56 patients (4%) with metastatic disease underwent surgery in 2019 to 2021. Palliative chemotherapy without a resection increased from 238 patients (9%) in 1989 to 1993 to 593 (40%) in 2019 to 2021 (Figure 2B). Since 2009, patients were also treated with targeted therapy, usually in combination with chemotherapy. In 2019 to 2021, 70 of 592 patients (12%) with metastatic disease receiving systemic chemotherapy were treated with targeted therapy and chemotherapy.

### Treatment Outcomes

**Figure 3.**
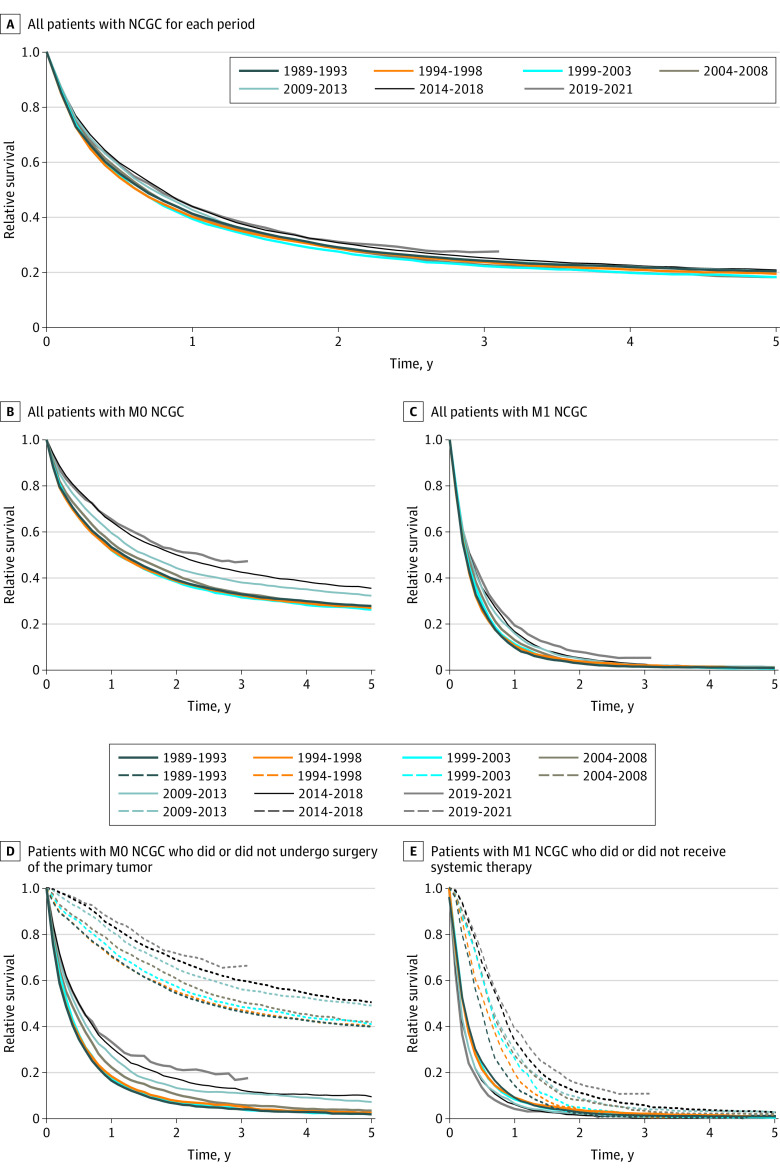
Relative Survival Dotted lines represent patients who underwent surgery (D) or received systemic therapy (E), solid lines represent patients who did not undergo surgery or receive systemic therapy, respectively. NCGC indicates noncardia gastric cancer.

Relative survival of all patients diagnosed with NCGC remained approximately the same, with a 5-year relative survival of 20% in all time periods. One-year relative survival for all patients increased from 41.3% (95% CI, 40.3%-42.3%) to 43.8% (95% CI, 41.9%-45.7%), and median survival increased from 8 months in 1989-1993 to 9 months in 2019-2021 (Figure 3A).

#### Nonmetastatic Disease

For patients with nonmetastatic disease, 5-year relative survival increased from 27.8% (95% CI, 26.5%-29.2%) in 1989 to 1993 to 35.5% (95% CI, 33.5%-37.6%) in 2014 to 2018. One-year survival increased from 53.3% (95% CI, 52.1%-54.6%) in 1989 to 1993 to 65.5% (95% CI, 62.8%-68.0%) in 2019 to 2021 (Figure 3B). For patients with nonmetastatic disease undergoing surgery, 5-year survival increased from 40.1% (95% CI, 38.3%-41.8%) in 1989 to 1993 to 50.6% (95% CI, 47.9%-53.3%) in 2014 to 2018. Median survival increased from 3 months in 1989 to 1993 to 6 months in 2019 to 2021 for patients with nonmetastatic disease not undergoing surgery, while for patients undergoing a resection, median overall survival was 29 months in 1989 to 1993 and could not be calculated for patients diagnosed between 2014 and 2018 because 51% of them were still alive at the end of our 5-year follow-up (Figure 3D).

#### Metastatic Disease

For patients with metastatic NCGC, 1-year relative survival increased from 9.9% (95% CI, 8.7%-11.1%) in 1989 to 1993 to 19.3% (95% CI, 17.2%-21.6%) in 2019 to 2021, but 3-year relative survival remained extremely poor at approximately 5% (95% CI, 3.6%-7.5%) for patients diagnosed between 2019 and 2021 (Figure 3C). For patients treated with systemic therapy for metastatic NCGC, relative 1-year survival increased gradually from 14.3% (95% CI, 10.4%-18.9%) in 1989 to 1993 to 39.2% (95% CI, 35.0%-43.4%) in 2019 to 2021. Median survival of these patients increased from 5 months in 1989 to 1993 to 10 months in 2019 to 2021 when systemic therapy was administered, and decreased from 3 months to 2 months when no systemic treatment was given (Figure 3E). In multivariable analysis, the RER of death decreased over time for all patients combined and for patients with nonmetastatic and metastatic disease separately (Table 2).

**Table 2.** Multivariable RER of Death for the Total Group and Patients With cM0 and cM1 Disease^a^

Period	Total group		cM0		cM1	
	RER (95% CI)	*P* value	RER (95% CI)	*P* value	RER (95% CI)	*P* value
1989-1993	1 [Reference]	NA	1 [Reference]	NA	1 [Reference]	NA
1994-1998	1.03 (0.99-1.06)	.13	1.00 (0.96-1.05)	.91	1.01 (0.95-1.07)	.73
1999-2003	1.05 (1.01-1.09)	<.01	1.02 (0.97-1.06)	.39	0.98 (0.92-1.04)	.44
2004-2008	1.01 (0.98-1.05)	.44	0.94 (0.90-0.99)	.02	0.91 (0.86-0.96)	<.01
2009-2013	0.95 (0.91-0.98)	<.01	0.80 (0.76-0.84)	<.001	0.82 (0.78-0.87)	<.001
2014-2018	0.91 (0.87-0.94)	<.001	0.69 (0.65-0.73)	<.001	0.78 (0.73-0.82)	<.001
2019-2021	0.92 (0.87-0.97)	<.01	0.65 (0.59-0.71)	<.001	0.73 (0.68-0.78)	<.001

Abbreviations: NA, not applicable; RER, relative excess risk.

^a^ Adjusted for age, sex, histology and Laurén classification. A decrease in RER can be observed from 2009 to 2013 for the entire population and patients with cM0 disease and from 2004 to 2008 for patients with cM1.

## Discussion

In this nationwide cohort study involving 47 014 patients diagnosed with NCGC between 1989 and 2021, a decreasing incidence was observed in line with the decrease seen in gastric cancer incidence worldwide.^26,27,28,29^ Over the decades, no changes in patient characteristics (ie, sex and age), but a significant change in various tumor characteristics, was seen. Some of these characteristics, eg, clinical staging, became more accurate as the unclassified categories (eg, Tx, Nx) decreased over time. For M stage, the increase in the proportion of patients with M1 disease is probably also caused by better staging techniques, leading to fewer patients erroneously considered as having M0 disease due to hidden metastatic disease. In the PLASTIC study, investigating the value of staging laparoscopy in patients with gastric cancer, in 19% of participants, peritoneal metastases were found that would otherwise have been overlooked. Considering that an increasing proportion of NCGC is of the diffuse type and that these cancers are known to metastasize mainly to the peritoneum, the increasing incorporation of staging laparoscopy in the diagnostic workup of these patients is an important improvement.^13,30^ For morphology, more diffuse adenocarcinomas were diagnosed over time. It is unlikely that this observation is due to technical or clinical improvements and is more likely to represent an actual increase in the proportion of diffuse adenocarcinomas, as was also seen in prior research.^31^

The observed decrease in incidence has been attributed to changes in dietary patterns, better food cooling techniques (eg, introduction of the refrigerator), and the reduction of *H pylori* prevalence. The decrease in incidence was stronger in men than in women, which might be due to a decrease in intestinal-type gastric cancers but not in diffuse-type cancers, with the latter being more common in female patients compared with male patients.^32^ Contrary to previous findings in the United States showing an increased incidence in young adults (<50 years), we did not observe this phenomenon in young adults in our data.^33^ It has been hypothesized that despite the substantial decrease in incidence of gastric cancer over the past decades, the increasing size and older age of the world population could result in an increase of 62% in absolute gastric cancer cases between 2020 and 2040 if current incidence rates stabilize.^18^

For the entire study population, unadjusted relative survival rates did not improve between 1989 to 1993 and 2019 to 2021, but they did improve for patients with nonmetastatic and metastatic disease separately. A possible explanation for this could be the Will Rogers phenomenon, which leads to a migration in stage-specific survival due to improved staging techniques, even though the survival of the individual patient has not actually changed. With the incorporation of guidelines recommending new staging techniques, such as diagnostic laparoscopy uncovering metastatic disease before it becomes clinically evident, an increasing number of patients is classified into higher stage disease, which leads, due to stage migration, to better survival outcomes both in the metastatic and nonmetastatic cancer group.

When adjusted for age, sex, histology, and Laurén classification in a multivariable regression model, survival did improve for the total population and for patients with nonmetastatic and metastatic disease separately. It is therefore hard to believe that the Will Rogers phenomenon is the only explanation for improvements in gastric cancer survival, also because considerable advances have been made in treatment for gastric cancer.^34^ In 2006, the MAGIC trial showed the superiority of perioperative chemotherapy plus surgery over surgery alone in potentially curable gastric cancer and even better results were seen in the FLOT4 study.^10,11^ The introduction of neoadjuvant chemotherapy may also have led to a decrease in patients undergoing surgery, because in a proportion of these patients, occult metastases will be uncovered during neoadjuvant treatment or preoperative restaging, leading to fewer unfavorable surgical procedures. Furthermore, since 2012, the centralization of gastric cancer surgery has been associated with fewer surgical complications, better quality of care, and improved survival.^35^

For metastatic gastric cancer, in the period under study, outcomes have not improved significantly in any chemotherapy-only trial. However, the introduction of trastuzumab, a monoclonal antibody targeting the human epidermal growth factor 2 (HER2), in 2010 has led to improved survival rates in a subgroup of patients, and second-line treatment with paclitaxel and ramucirumab showed superior survival compared with paclitaxel alone.^36,37^ The development of novel systemic therapies, such as targeted therapies, are reflected in our data by the increase of relative survival since approximately 2009 to 2013 for patients with metastatic disease, even though the proportion of patients receiving targeted therapy is small. Further investigation of treatment trends in the upcoming years will be interesting, because of the development of these novel systemic treatment options, such as targeted therapies, and immune therapy options, such as targeted therapies and immunotherapy.^38^

Another reason to assume that treatment advances might have improved survival outcomes in NCGC is the observed shift from mainly intestinal type adenocarcinomas toward more diffuse-type adenocarcinomas over the past decades. According to previous findings, diffuse-type gastric cancers have an unfavorable prognosis compared with the intestinal type.^39^ If no true advances in treatment had been made, this shift should have led to a decrease in survival purely based on the higher proportion of diffuse-type cancers. However, when corrected for the Laurén classification in a multivariable regression model, the RER of death decreased in the total population and for patients with nonmetastatic and metastatic disease.

### Limitations

This study has limitations. The population-based design included more than 47 000 patients, and the broad selection criteria ensured the minimization of selection bias. However, for the comparison between survival of patients who did or did not undergo surgery or chemotherapy, possible immortal time bias could not entirely be avoided. However, the population-based design of this study also introduced certain limitations. For example, for most patients in our cohort, no information was available on reasons why certain treatment choices were made, and no follow-up data on recurrence or progression of disease was available. Also, for patients with metachronous metastatic disease, only information about the primary diagnosis and treatment thereof is registered. Therefore, all information about metastatic gastric cancer in this article comprises only synchronous metastatic disease. Another challenge was the fact that it was not easy to select a classification system that could be used over the entire period of the study, because the TNM classification systems in clinical practice have changed several times in the past 32 years.

## Conclusions

In this cohort study of more than 47 000 patients with NCGC, our data showed a decrease in incidence, a shift in treatment choices, and an increase in survival among patients with nonmetastatic and metastatic NCGC over the past 32 years in the Netherlands. However, despite the decreasing incidence, prognosis remains poor, and gastric cancer remains the fourth most common cause of cancer deaths.^1^ Therefore, the search for more effective surveillance and treatment strategies should be continued.

## Notes

Supplement.
Data Sharing Statement
