# Supplementary material for: Incidence, Stage, Treatment, and Survival of Noncardia Gastric Cancer
Source: JAMA Netw Open. 2023 Aug 21;6(8):e2330018. doi: 10.1001/jamanetworkopen.2023.30018 (PMC10442714; doi:10.1001/jamanetworkopen.2023.30018)
Supplement: Supplement. — Data Sharing Statement [file jamanetwopen-e2330018-s001.pdf]

Data Sharing Statement

Data

Data available: Yes
Data types: Deidentified participant data
How to access data: Data can be made available by Netherlands Comprehensive Cancer Organisation upon justified request. gegevensaanvraag@iknl.nl
When available: With publication

Supporting Documents

Document types: None

Additional Information

Who can access the data: researchers whose proposed use of the data has been approved
Types of analyses: for any justified purpose
Mechanisms of data availability: With a signed and approved data acces agreement
